# Effects of Ethanol Exposure During Adolescence or Adulthood on Locomotor Sensitization and Dopamine Levels in the Reward System

**DOI:** 10.3389/fnbeh.2020.00031

**Published:** 2020-03-06

**Authors:** Priscila Fernandes Carrara-Nascimento, Lucas Barbosa Hoffmann, Jorge Camilo Flório, Cleopatra Silva Planeta, Rosana Camarini

**Affiliations:** ^1^Departamento de Farmacologia, Instituto de Ciências Biomédicas, Universidade de São Paulo, São Paulo, Brazil; ^2^Departmento de Patologia, Escola de Medicina Veterinária, Universidade de São Paulo, São Paulo, Brazil; ^3^Laboratório de Neuropsicofarmacologia, Faculdade de Ciências Farmacêuticas, Universidade Estadual Paulista, Araraquara, Brazil

**Keywords:** ethanol, adolescence, prefrontal cortex, striatum, nucleus accumbens, mice

## Abstract

Behavioral sensitization is a process of neuroadaptation characterized by a gradual increase in motor behaviors. The major neural substrates involved in the behavioral sensitization lie on the dopaminergic mesocorticolimbic pathway, which is still under development during adolescence. To investigate age-differences in ethanol behavioral sensitization and dopamine levels in distinct brain regions of the reward system, adolescent and adult mice were repeatedly pretreated with saline or ethanol (2.0 g/kg i.p.) during 15 consecutive days and challenged with saline or ethanol 5 days after pretreatment. Dopamine and its metabolites were measured in tissue samples of the prefrontal cortex (PFC), nucleus accumbens (NAc) and striatum by HPLC analysis. While repeated ethanol administration resulted in the development of locomotor sensitization in both adult and adolescent mice, only the adults expressed sensitization to a subsequent ethanol challenge injection. Neurochemical results showed reduced dopamine levels in adolescents compared to adults. Specifically, mice pretreated with ethanol during adolescence displayed lower dopamine levels in the PFC compared to the respective adult group in response to an ethanol challenge injection, and preadolescent mice exhibited lower dopamine levels in the NAc following an acute ethanol treatment compared to adults. These findings suggest that adolescent mice are not only less sensitive to the expression of ethanol-induced sensitization than adults, but also show lower dopamine content after ethanol exposition in the PFC and NAc.

## Introduction

Alcohol is a widely abused substance in human society, which is associated with economic, health and family costs. Adolescents who start drinking at the age of 14 or younger show a higher prevalence of lifetime alcohol use disorders than those who start drinking at ages 20 or older (Grant and Dawson, 1997), suggesting that early consumption of alcohol increases the vulnerability to addiction.

Adolescents show a characteristic pattern of behavioral responses to ethanol that differs from adults. Adolescent rodents are less sensitive to ethanol’s sedative effects and to ethanol behavioral sensitization as compared to adults, but show higher sensitivity to its appetitive effects (Faria et al., 2008; Pautassi et al., 2008). The profile of neurochemical responses to ethanol in adolescents is also distinct from adults (Pascual et al., 2009; Guerri and Pascual, 2010; Carrara-Nascimento et al., 2011; Mishra and Chergui, 2013; Crews et al., 2016).

The protracted development of the mesocorticolimbic dopamine pathway explains, in part, typical characteristics of adolescents, such as cognition immaturity, impulsive behavior, novelty and reward-seeking and risky decision making (for review, see Spear, 2000). The prefrontal cortex (PFC) undergoes important developmental changes during adolescence in humans and rats (Insel et al., 1990; Giedd et al., 1999) and dopaminergic inputs to this region have inhibitory functions, enabling control of attention, motivation and decision making.

The limbic system undergoes important and determinant maturational changes for the transition from infancy to adulthood. Adolescent rats (at PND 30) exhibited lower basal levels of dopamine compared to adults in tissue samples of the striatum (Teicher et al., 1993) and reduced storage pool of releasable dopamine in this region (Stamford, 1989). Although similar dopamine basal levels were found in tissue samples of nucleus accumbens (NAc) and frontal cortex between adolescent and adult rats (Teicher et al., 1993), microdialysis studies demonstrated peaks at PND 45 compared to younger or older rats in the NAc (Badanich et al., 2006; Philpot et al., 2009). In general, basal dopamine efflux from NAc obey an inverted U-shaped curve (Philpot et al., 2009) and repeated ethanol exposure during adolescence alters the pattern of basal dopamine levels (Badanich et al., 2007; Pascual et al., 2009). These changes may be determinant to promote reward-seeking behavior (Pascual et al., 2009; Alaux-Cantin et al., 2013) since ethanol exposure during adolescence alters goal-directed behavior and judgment toward poor decision-making and risk-taking behavior (Goudriaan et al., 2007; see Alfonso-Loeches and Guerri, 2011, for review). In fact, stimulant effects of ethanol are determined mostly by its actions on the synthesis, release and turnover of dopamine of dopaminergic neurons (Fadda et al., 1980; Di Chiara and Imperato, 1985; Brodie et al., 1999; Bassareo et al., 2017), whose actions are responsible for its reinforcing effects.

Behavioral sensitization is a progressive increase in behavioral responses to drugs or stress that represents a neuroplastic outcome of enduring events occurring in the dopaminergic mesolimbic pathway (Wise and Bozarth, 1987; Robinson and Berridge, 2000). Behavioral sensitization is conceptualized into two phases: initiation and expression. The initiation or development of sensitization reflects the immediate events occurring in the ventral tegmental area (VTA), while the expression reveals long-term consequences of the initial neural alterations after cessation of the treatment (Kalivas and Stewart, 1991). In general, the neural changes underlying the expression of sensitization require a withdrawal period. Furthermore, there is evidence for the overlapping of neural circuitries responsible for behavioral sensitization and reinstatement (Steketee and Kalivas, 2011), suggesting a link between the expression of sensitization and relapse. Among the neural changes underlying behavioral sensitization, the mesocorticolimbic dopamine system is critically involved (Steketee and Kalivas, 2011). Studies focused on dopaminergic underpinnings of chronic ethanol consumption and withdrawal have shown that repeated and continuous exposure to ethanol followed by withdrawal periods results most often in a hypodopaminergic state (Diana et al., 1996), although the hyperdopaminergic state has been reported in protracted abstinence (Hirth et al., 2016). An electrophysiological study showed that ethanol-induced behavioral sensitization induced enhancement of the basal spontaneous firing rate of dopamine neurons in the VTA (Didone et al., 2016).

A different profile of cocaine behavioral sensitization and dopaminergic neurochemical sensitization were found in adolescent mice compared to their adult counterparts. Adolescents exhibited greater behavioral sensitization and lower sensitization to dopamine overflow compared to adults (Camarini et al., 2008), which was associated with a higher expectancy of the drug in adolescents. However, contrary to what was observed with cocaine (Camarini et al., 2008; Valzachi et al., 2013), adolescent mice are less sensitive to ethanol-induced locomotor sensitization than adults (Stevenson et al., 2008; Carrara-Nascimento et al., 2011; Camarini and Pautassi, 2016). Despite the existing literature on alcohol-induced changes in the dopaminergic system of adolescents, studies do not correlate ethanol behavioral sensitization with alterations in dopamine levels in brain regions involved in the phenomenon.

The present study aimed to evaluate locomotor behavioral responses to acute and repeated ethanol in adolescent and adult mice and quantify their dopamine and its metabolites levels in tissue homogenates of specific brain regions related to the reward system (PFC, striatum, and NAc).

## Materials and Methods

### Animals

Adolescent and adult male Swiss mice were obtained from the Animal Facility of the Department of Pharmacology of the Institute of Biomedical Sciences at the Universidade de São Paulo, Brazil. Mice were housed in groups of five in standard Plexiglas cages (30 cm × 20 cm × 12.5 cm) in a colony room with controlled lighting (12:12 light/dark cycle; lights on from 7:00 AM to 7:00 PM) and temperature (22 ± 2°C) conditions. All mice were allowed to adapt to the colony room for at least 7 days before the beginning of the experiments. At the beginning of the experiments, adolescents were PND 28–30 and adults, PND 68–70. Food and water were provided *ad libitum*. All procedures were approved by the Ethics Committee on Animal Use (Comitê de Ética no Uso de Animais—CEUA—Protocol #81/2013) of the Institute of Biomedical Sciences of the Universidade de São Paulo.

### Drugs

Ethanol (Merck do Brasil, Rio de Janeiro, RJ, Brazil) solution at 20% was prepared from 95% (v/v) ethanol and administered at a dose of 2.0 g/kg *via* intraperitoneal injections. This dose was achieved by administering 0.125 ml per 10 g of body weight. Control mice were administered isovolumetric injections of the vehicle solution (0.9% v/v saline). The dose and treatment regimen were chosen based on previous studies that show reliable behavioral sensitization in Swiss male mice (for review, see Camarini and Pautassi, 2016). For instance, initiation and expression of sensitization depend on a number of factors: number of injections and interval between them, ethanol dose, species, strain, sex, among others. In Didone et al. (2008), lower ethanol doses (1.5–2.0 g/kg) resulted in better expression during the first 10–15 min after ethanol injection than higher doses (2.5–3.0 g/kg).

### Apparatus

The locomotor activity was assessed in a cylindrical open-field arena (40 cm diameter and 35 cm high). A video camera, placed above the apparatus and connected to a computer located outside the experimental room, recorded the trials. The apparatus was cleaned with a 5% ethanol/water solution between each trial. Injections and locomotor activity assessments were always carried out between 9:00 AM and 11:30 AM.

### Behavioral Sensitization Procedure

First, animals were habituated to the injections and open-field apparatus for two consecutive days (Habituation days: H1 and H2). Mice were injected intraperitoneally (i.p.) with saline (0.9% w/v sodium chloride, SAL) and placed in the open-field for 5 min, 5 min after the injection.

The experimental design of the behavioral sensitization consisted of a phase of initiation of locomotor sensitization (15 days), followed by an abstinence period (5 days) and then, by a test day, when the expression of sensitization was evaluated.

On the next day after the last habituation session, adolescent and adult mice were distributed into the experimental groups (saline and ethanol) for the initiation of behavioral sensitization. One-half of mice of each age group received daily i.p. injections of saline, while the other half was treated with 2.0 g/kg ethanol (20% v/v ethanol in saline), resulting in four experimental groups: Adolescent-SAL (*n* = 20), Adolescent-EtOH (*n* = 20), Adult-SAL (*n* = 20) and Adult-EtOH (*n* = 20). The treatment lasted 15 days and the locomotor activity was quantified only on days 1, 8 and 15. Animals were exposed to the open-field arena only during recording days, as previously described in Camarini et al. (2008). The animals’ locomotor activity (distance traveled in cm) was assessed during a 5 min-period, 5 min after saline or ethanol injection. This period (5–10 min after injection) fits in the time window of the peak of the acute stimulation and the locomotor sensitization effect of ethanol (Phillips et al., 1995; Legastelois et al., 2015), Moreover, this procedure minimizes any association of discomfort due to ethanol injection with the apparatus.

After 5 days of abstinence, on experimental day 21, mice were tested for the expression of ethanol sensitization. Half of each experimental group was challenged with 2.0 g/kg ethanol, while the other half was injected with a saline injection, establishing eight experimental groups: Adolescent-SAL/SAL (*n* = 10), Adolescent-SAL/EtOH (*n* = 10), Adolescent-EtOH/SAL (*n* = 10), Adolescent-EtOH/EtOH (*n* = 10), Adult-SAL/SAL (*n* = 10), Adult-SAL/EtOH (*n* = 10), Adult-EtOH/SAL (*n* = 10) and Adult-EtOH/EtOH (*n* = 10). The expression of sensitization was conducted in the adolescent group on PND 50–52, and in the adult group on PND 90–92.

Mice were euthanized by cervical dislocation 40 min after the injections since peaks of striatal extracellular dopamine after systemic injection of 2.0 g/kg ethanol is reached around 40 min (Bosse and Mathews, 2011).

### Quantification of Dopamine and Metabolites in the Brain Tissue

The brains were removed, cooled on ice, and three brain regions were dissected, based on the mouse brain atlas (Paxinos and Franklin, 2001). Brains were placed in a mouse brain matrix (ASI-Instruments^®^, Houston, TX, USA), used to provide coronal brain sections. The brains were cut and mounted on slides (SuperFrost Plus, Thermo Fisher Scientific, MA, USA). Brain punches (1.2 mm or 1.0 mm) of the PFC, NAc, and striatum were obtained with micro punches (Harris Micro-Punch, Ted Pella). Specifically, the punched area in the frontal cortex was focused in the mPFC. The brain tissues were frozen in liquid nitrogen and maintained at −80°C for later quantification of dopamine and the metabolites DOPAC (3,4-Dihydroxyphenylacetic acid) and HVA (homovanillic acid).

The tissues (PFC, NAc and striatum) were homogenized and sonicated in 0.1 M perchloric acid solution, prepared by adding 8.68 mL of concentrated perchloric acid, 200 mg of sodium metabisulphite—Na_2_S_2_O_5_—and 200 mg of EDTA in 1.0 L of MilliQ ultrapure water, containing 28.9 ng/mL of dihydroxy-benzylamine (DHBA). The homogenates were centrifuged at 10,000 rpm for 20 min at 4°C. At the time of homogenization, the tissues were weighed (still frozen) immediately before adding the perchloric acid solution. For each mg of tissue, 15 μl of the perchloric acid solution with DHBA was added. Dopamine, DOPAC and HVA were measured by high-performance liquid chromatography with an electrochemical detector (HPLC model LC20 AD, Shimadzu, Japan and Detector Antec Decade sdc VT 03 electrochemical Flow Cell), with a C-18 column (Shimpak; ODS, 15 cm, Kyoto, Japan), and an integrator (model 20AC Chromatopac; Shimadzu). The limit of detection was 0.02 ng for DA, DOPAC and HVA.

### Statistical Analysis

Details of the statistical test and sample size for each experiment are summarized in Supplementary Table S1.

The locomotor activity evaluated throughout the days (habituation days, H1 and H2 and treatment days, D1, D8 and D15) was analyzed by three-way ANOVAs, considering three factors [Age (adolescent and adult)] × [Treatment (saline or ethanol)] × Days as repeated measures. This analysis allowed to compare significant differences between adolescents and adults. When appropriate, two-way ANOVAs were followed-up to analyze differences within each age, considering treatment and days as repeated measures. Data from the locomotor activity measured on the challenge day, when mice pretreated with saline or ethanol were challenged with saline or ethanol (Challenge Day), were analyzed by a three-way ANOVA, considering three factors [Age (adolescent and adult)] × [Pretreatment (saline or ethanol)] × [Challenge injection (saline or ethanol)]. Two-way ANOVAs [Pretreatment (saline or ethanol)] × [Challenge injection (saline or ethanol)] were conducted for each age group.

Dopamine and metabolites levels were analyzed by a three-way ANOVA considering three factors [Age (adolescent and adult)] × [Pretreatment (saline or ethanol)] × [Challenge injection (saline or ethanol)]. Two-way ANOVAs [Pretreatment (saline or ethanol)] × [Challenge injection (saline or ethanol)] were conducted for each age group.

ANOVAs were followed by Tukey HSD test as *post hoc* when significant interactions of factors were detected. A Bonferroni-corrected multiple comparison test was used when only significant main effects were found.

Levene’s test was employed to test homogeneity of variance and assumptions for normal distribution was tested with the Shapiro–Wilks test. In case the analyses were found not normally distributed or due to unequal variance, Kruskal–Wallis was used to assess differences among the groups. Mann–Whitney test was used for pairwise comparisons.

The data are presented as mean ± SEM, except for the data from PFC (non-parametric data), which are expressed as the median values and interquartile range. Statistical significance was considered when *p* < 0.05. The program SPSS Statistics for Windows was used to analyze the data (SPSS Statistics, Armonk, NY, USA: IBM Corporation, Armonk, NY, USA).

## Results

### Behavioral Sensitization

#### Habituation Days

**Figure 1:**
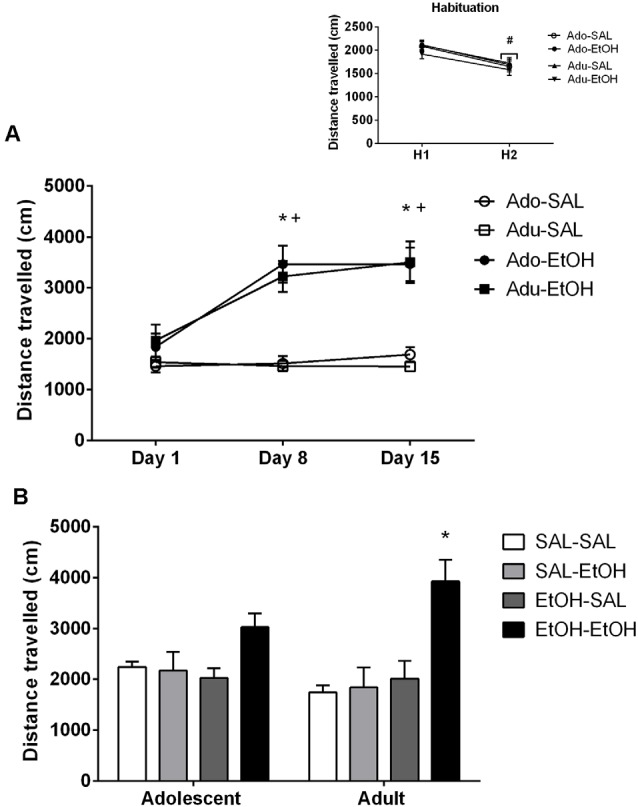
Locomotor activity of adolescent and adult mice. **(A)** Adolescent (Ado) and adult (Adu) mice were pretreated with saline (SAL) or ethanol (EtOH) during 15 consecutive days and the locomotor activity was assessed on Days 1, 8 and 15. *Denotes significant differences in locomotor activity from Day 1; ^+^denotes significant differences in locomotor activity of ethanol-treated mice compared to saline-treated mice. The smaller figure shows the locomotion of mice injected with saline during two consecutive days (habituation days), ^#^locomotion of each group on the second day of habituation (H2) was lower than on the first day they were exposed to the open-field (habituation day: H1). **(B)** Locomotor activity assessed on Day 21 in mice challenged with saline (-SAL) or ethanol (-EtOH) 5 days after pretreatment with saline (SAL-) or ethanol (EtOH-). *Denotes greater locomotor activity compared to all the other groups within the same age group. The values are expressed as mean ± SEM.

Analysis of the data by a three-way ANOVA revealed a main effect of days (day effect, *p* < 0.001; Figure 1A). Bonferroni-corrected comparisons indicated that the locomotor activity of each group on H2 (second day of habituation) was significantly reduced compared to that on H1 (first day of habituation), consistent with habituation to the apparatus.

#### Development of Ethanol Behavioral Sensitization

Analysis of the data by a three-way ANOVA revealed main effects of treatment (*p* < 0.001), days (*p* < 0.001) and a treatment × days interaction (*p* < 0.001; Figure 1A). Main effect of age was non-significant (*p* = 0.82). Two-way ANOVAs were applied separately to the locomotor activity data from each age. Analysis of the adolescent data revealed main effects of treatment (*p* < 0.001), days (*p* < 0.001) and a treatment × days interaction (*p* < 0.001; Figure 1A). Tukey HSD test revealed that adolescent mice treated with ethanol exhibited higher locomotion than saline counterparts on days 8 and 15). The locomotion of ethanol-treated mice was greater on days 8 and 15 compared to day 1 (first session). There were no significant differences in the locomotor activity of mice treated with saline. Analysis of the adult data revealed main effects of treatment (*p* < 0.001), days (*p* < 0.001) and a treatment × days interaction (*p* < 0.001; Figure 1A). Tukey HSD test revealed similar results as those observed in the adolescent group.

#### Expression of Ethanol Behavioral Sensitization

The locomotion of mice pretreated with saline or ethanol and challenged with saline or ethanol is depicted in Figure 1B. Analysis of the data by a three-way ANOVA revealed main effects of pretreatment (*p* < 0.01), challenge injection (*p* < 0.01) and a pretreatment × challenge injection interaction (*p* < 0.01; Figure 1B). Main effect of age was non-significant (*p* = 0.94). Tukey HSD test performed to analyze pretreatment × challenge injection interaction revealed that mice pretreated with ethanol and challenged with ethanol (EtOH-EtOH) displayed greater locomotor activity than those mice challenged with saline (EtOH-SAL). Two-way ANOVAs were followed-up to analyze differences within each age group. Analysis of the adolescent data revealed a pretreatment × challenge injection interaction (*p* < 0.05). Tukey HSD test did not reveal significant differences among adolescent groups, except for a trend between EtOH-SAL and EtOH-EtOH (*p* = 0.051). Analysis of the adult data revealed a pretreatment effect (*p* < 0.01), a main effect of challenge injection (*p* < 0.01) and a pretreatment × challenge injection interaction (*p* < 0.05). Tukey HSD test revealed that adult mice pretreated and challenged with ethanol (EtOH-EtOH) displayed greater locomotor activity compared to all the other groups (all p’s < 0.001), suggesting a robust expression of behavioral sensitization.

### Dopamine and Metabolites Quantification

Dopamine and metabolites were quantified in mice at PND 50–52 (Adolescent group) and at PND 90–92 (Adult group).

#### Prefrontal Cortex

**Figure 2:**
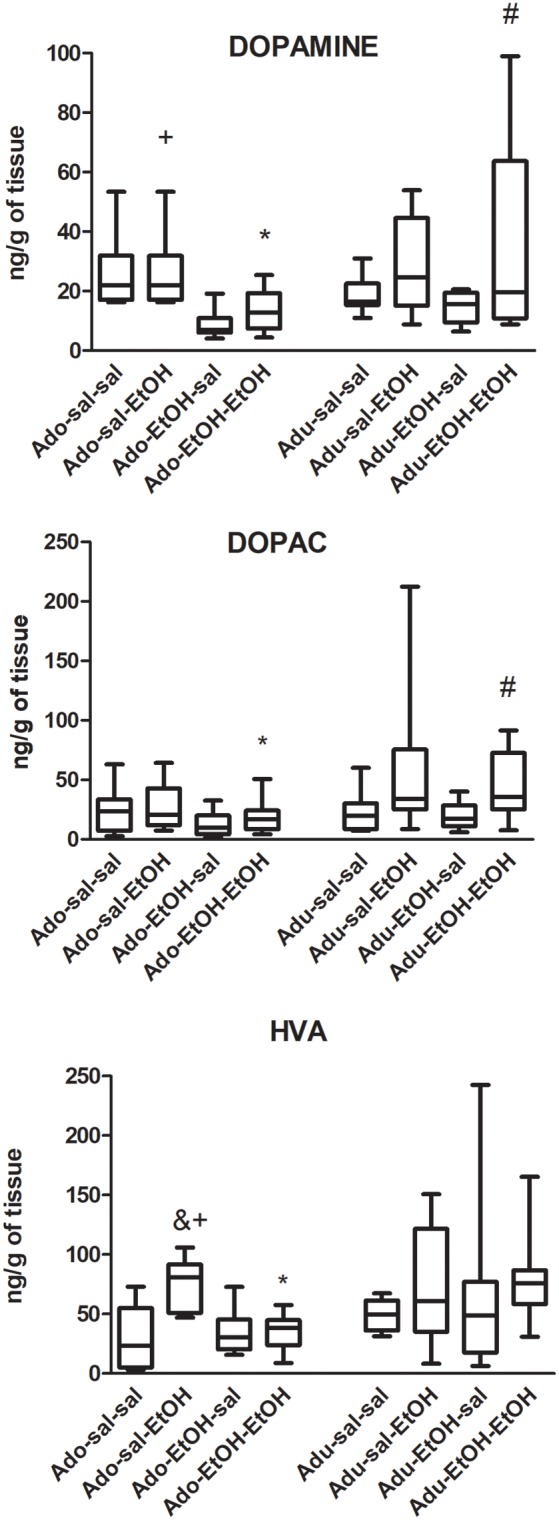
Dopamine, DOPAC and HVA levels in the prefrontal cortex (PFC) of adolescent and adult mice pretreated with saline (SAL-) or ethanol (EtOH-) and challenged with saline (-SAL) or ethanol (-EtOH). *Adolescent group differ from the respective adult group, within the same treatment group. ^#^Different from mice pretreated with ethanol and challenged with saline (EtOH-SAL) within the same age group, ^+^differ from mice pretreated and challenged with ethanol (EtOH-EtOH), within the same age group, ^&^different from the respective control group, within the same age group (SAL-SAL). Data are expressed as median and interquartile range.

The results of DA, DOPAC and HVA levels are depicted in Figure 2. Number of samples/group = 10. The non-parametric Kruskal–Wallis test was used for the analysis of dopamine and metabolites, and Mann–Whitney’s test was used to assess the differences between experimental groups.

##### Dopamine

*Pair-wise* comparisons revealed that dopamine levels were lower in adolescent mice pretreated and challenged with ethanol (EtOH-EtOH) compared to the respective adult group (*U* = 24, *Z* = −1.97, *p* < 0.05) and to the adolescent mice pretreated with saline and challenged with ethanol (*U* = 5; *Z* = 3.4, *p* < 0.05). Adult mice pretreated and challenged with ethanol (EtOH-EtOH) exhibited higher dopamine levels than those pretreated with ethanol and challenged with saline (EtOH-SAL; *U* = 24, *Z* = −1.9; *p* < 0.05).

##### DOPAC

*Pair-wise* comparisons revealed that DOPAC levels were lower in adolescent mice pretreated and challenged with ethanol (EtOH-EtOH) compared to the respective adult group (*U* = 24, *Z* = −2.00, *p* < 0.05). Adult mice pretreated and challenged with ethanol (EtOH-EtOH) exhibited higher DOPAC levels than those pretreated with ethanol and challenged with saline (EtOH-SAL; *U* = 22, *Z* = −2.12; *p* < 0.05).

##### HVA

*Pair-wise* comparisons revealed that HVA levels were lower in adolescent mice pretreated and challenged with ethanol (EtOH-EtOH) compared to the respective adult group (*U* = 12, *Z* = −2.87, *p* < 0.001). Adolescent mice pretreated with saline and challenged with ethanol (SAL-EtOH) displayed higher HVA levels compared to controls (SAL-SAL; *U* = 10, *Z* = −3.02; *p* < 0.001) and to mice repeatedly treated with ethanol and challenged with ethanol (EtOH-EtOH; *U* = 5, *Z* = 3.4, *p* < 0.001).

#### Nucleus Accumbens

**Figure 3:**
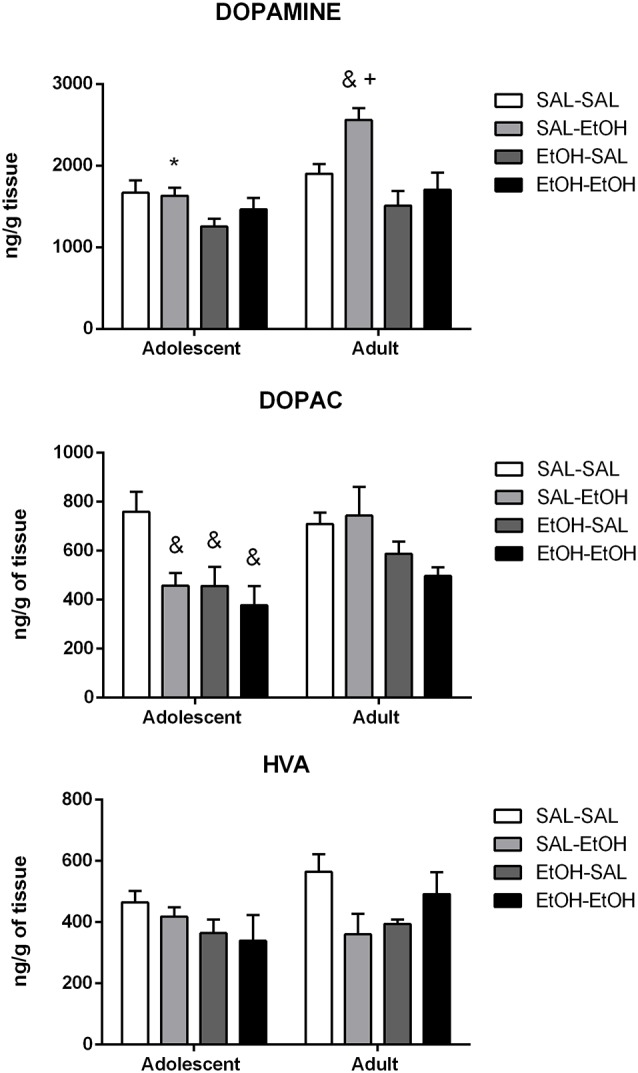
Dopamine, DOPAC and HVA levels in the nucleus accumbens (NAc) of adolescent and adult mice pretreated with saline (SAL-) or ethanol (EtOH-) and challenged with saline (-SAL) or ethanol (-EtOH). *Adolescent mice differ from the respective adult mice, within the same treatment group, ^+^different from ethanol-pretreated mice challenged with ethanol (EtOH-EtOH), within the same age group, ^&^different from the respective control group, within the same age group (SAL-SAL). The values are expressed as mean ± SEM.

The results are shown in Figure 3. Number of samples/group = 8. Few samples were lost because of analytical failure.

##### Dopamine

Analysis of the data by a three-way ANOVA revealed main effects of age (*p* < 0.001), pretreatment (*p* < 0.001) and challenge injection (*p* < 0.01). Multiple comparisons with Bonferroni correction showed that the difference was between the age groups repeatedly treated with saline and challenged with ethanol (SAL-EtOH).

Two-way ANOVAs were used to analyze differences within each age. Although ANOVA has revealed the main effect of pretreatment (*p* < 0.05), *post hoc* pairwise comparisons did not show significant differences among adolescent groups. Analysis of the adult data by a two-way ANOVA revealed main effects of pretreatment (*p* < 0.001) and challenge injection (*p* < 0.05). Multiple comparisons with Bonferroni correction indicated that mice pretreated with saline and challenged with ethanol (SAL-EtOH) exhibited higher dopamine levels compared to their control group (SAL-SAL) and to mice repeatedly treated with ethanol and challenged with ethanol (EtOH-EtOH).

##### DOPAC

Analysis of the data by a three-way ANOVA revealed main effects of age (*p* < 0.05), pretreatment (*p* < 0.001) and challenge injection (*p* < 0.05). However, planned pairwise comparisons with Bonferroni correction did not show significant differences between age groups.

Two-way ANOVAs were used to analyze differences within each age. Analysis of the adolescent data by a two-way ANOVA revealed main effects of pretreatment (*p* < 0.05) and challenge injection (*p* < 0.05). Multiple comparisons with Bonferroni correction revealed lower DOPAC levels in mice treated with acute ethanol (SAL-EtOH) or pretreated with ethanol (EtOH-SAL or EtOH-EtOH) compared to their control group (SAL-SAL). Two-way ANOVA performed on the adult data revealed a main effect of pretreatment (*p* < 0.05). However, pairwise comparisons with Bonferroni correction did not show significant differences between age groups.

##### HVA

Analysis of the data by a three-way ANOVA did not find statistically significant differences between adolescent and adult groups (age effect, *p* > 0.05).

Two-way ANOVAs were used to analyze differences within each age. No statistically significant differences were found among groups for adolescents or for adults.

#### Striatum

**Figure 4:**
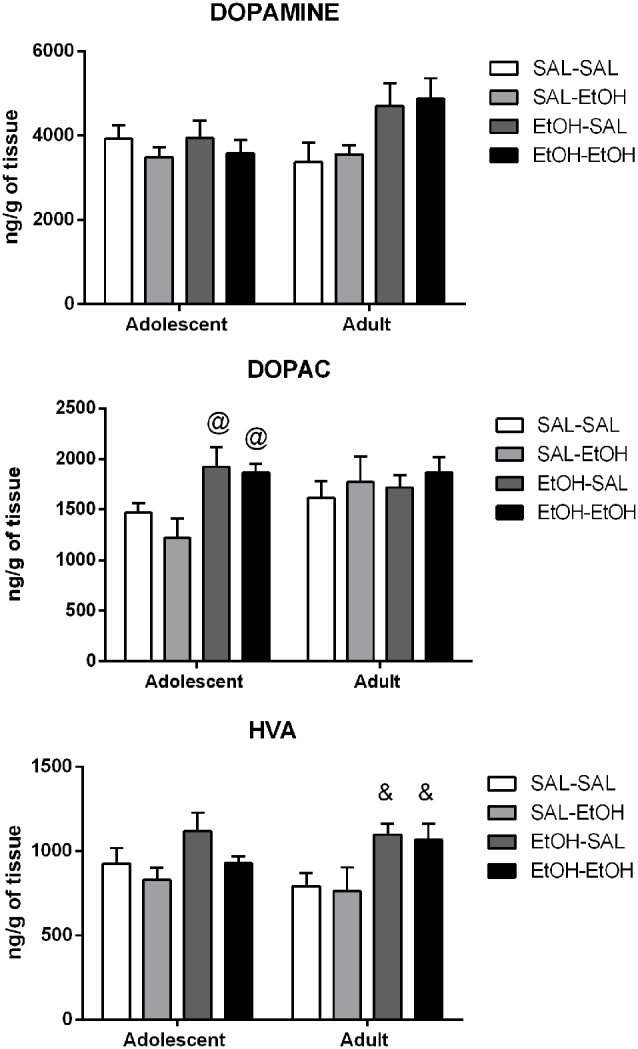
Dopamine, DOPAC and HVA levels in the striatum of adolescent and adult mice pretreated with saline (SAL-) or ethanol (EtOH-) and challenged with saline (-SAL) or ethanol (-EtOH). ^@^Different from the saline-pretreated mice challenged with ethanol, within the same age group (SAL-EtOH), ^&^different from the respective control group, within the same age group (SAL-SAL). The values are expressed as mean ± SEM.

The results are shown in Figure 4. Number of samples/group = 10.

##### Dopamine

Analysis of the data by a three-way ANOVA did not find statistically significant differences between adolescent and adult groups (age effect, *p* > 0.05).

Two-way ANOVAs were used to analyze differences within each age. No statistically significant differences were found among adolescent groups. Two-way ANOVA performed on the adult data revealed a main effect of pretreatment (*p* < 0.05). However, pairwise comparisons revealed only a statistically non-significant trend to increased dopamine levels in ethanol-pretreated adult mice challenged with ethanol compared to their controls (SAL-SAL; *p* = 0.06).

##### DOPAC

Analysis of the data by a three-way ANOVA did not find statistically significant differences between adolescent and adult groups (age effect, *p* > 0.05).

Two-way ANOVAs were used to analyze differences within each age. Analysis of the adolescent data by a two-way ANOVA revealed a main effect of pretreatment (*p* < 0.001). Pairwise comparisons with Bonferroni correction revealed increased DOPAC levels in ethanol-pretreated adolescent mice challenged with saline (EtOH-SAL) or ethanol (EtOH-EtOH) compared to those mice acutely treated with ethanol (SAL-EtOH). No statistically significant differences were found among adult groups.

##### HVA Levels

Analysis of the data by a three-way ANOVA did not find statistically significant differences between adolescent and adult groups (age effect, *p* > 0.05).

Two-way ANOVAs were used to analyze differences within each age. No statistically significant differences were found among adolescent groups. Two-way ANOVA performed on the adult data revealed a main effect of pretreatment (*p* < 0.01). Pairwise comparisons with Bonferroni correction revealed that ethanol-pretreated adult mice challenged with saline (EtOH-SAL) or ethanol (EtOH-EtOH) displayed higher HVA levels than their control group (SAL-SAL).

## Discussion

The present study suggests that adolescent mice are less sensitive to the expression of locomotor sensitization to ethanol as compared to adults, which has been previously demonstrated by other studies (Faria et al., 2008; Stevenson et al., 2008; Quoilin et al., 2012; Soares-Simi et al., 2013; Carrara-Nascimento et al., 2014, 2017) and highlights differential levels of dopamine in brain regions involved in the rewarding circuitry.

The attenuated effect on sensitization expression in adolescents could be admitted to a possible ceiling effect in response to 2.0 g/kg ethanol. However, this is unlikely because mice can sensitize to higher doses of ethanol, like 2.5 or 4.0 g/kg (Stevenson et al., 2008; Quoilin et al., 2012). We also questioned if ethanol exposure during adolescence could result in metabolic changes and alter the pharmacological profile to ethanol responses. Although this possibility cannot be discarded, previous studies have investigated the consequences of ethanol exposure during adolescence on blood alcohol concentration (BEC) and aldehyde dehydrogenase activity (ALDH). No differences in BECs were found between adolescents and adults following repeated ethanol administration, despite the differential magnitude in behavioral sensitization between them (Stevenson et al., 2008; Quoilin et al., 2012). Also, chronic ethanol pretreatment in adolescent and adult mice did not result in differential ALDH activity, although it had an impact on ethanol consumption patterns (Carrara-Nascimento et al., 2017). Despite these pieces of evidence, a study conducted by Linsenbardt et al. (2009) demonstrated that adolescent mice exhibited lower BEC than adults after acute and chronic administration of 4.0 g/kg ethanol.

Age-dependent differences in behavioral responses are not limited to alcohol. For instance, adolescent rats exhibited lower sensitivity to kappa agonists compared to adults (Anderson et al., 2013). Interestingly, ethanol and opioids share mechanisms of action to increase dopamine release (Lindholm et al., 2007).

Besides the reduced sensitivity to expression of ethanol sensitization, the main findings of the dopamine analysis in homogenates of brain regions suggest lower content after ethanol exposition in the PFC, and NAc of adolescents compared to adults. Although it is reasonable to speculate that adolescents have achieved their ceiling effect with 2.0 g/kg ethanol dose, our study was conducted in tissue samples instead of dialysates, and it has been reported that 2.0 g/kg ethanol enhances extracellular levels of dopamine by only 40% from the basal levels (Yim et al., 2000).

Acute ethanol has direct effects on dopaminergic neurons at VTA, which can alter dopamine release and its activity in the PFC (Harrison et al., 2017). Previous studies have demonstrated increased dopamine levels in the PFC after acute i.v infusion or posterior VTA administration of ethanol in adult rats (Ding et al., 2011; Schier et al., 2013). In the present study, we did not find significant increases in dopamine levels after acute ethanol in adolescent or adult mice, albeit dopamine and DOPAC levels were elevated in adult mice repeatedly treated with ethanol following a challenge ethanol injection compared to the respective adult group challenged with saline. These results are particularly interesting because of the contribution of dopaminergic neurotransmission in the PFC to behavioral sensitization (Bjijou et al., 2002). Moreover, adolescent mice repeatedly treated and challenged with ethanol exhibited lower dopamine levels in the PFC compared to the respective adult group and to adolescent mice that received an acute ethanol injection. Similar age differences were found for DOPAC and HVA results. The findings also suggest the development of a dopaminergic tolerance to repeated ethanol treatment in the adolescent group. The PFC receives dopaminergic projections into the prelimbic and infralimbic regions that are involved in goal-directed behaviors (Hitchcott et al., 2007), cognitive control processes, motivation, and in responses to salient and relevant stimuli (Ott and Nieder, 2019). We have previously demonstrated that repeated exposure to ethanol during adolescence lowered Fos and Egr-1 protein expression (Faria et al., 2008) and cAMP response element-binding protein (CREB)-binding activity in the PFC (Soares-Simi et al., 2013) compared to adults. The dopamine D_1_ signaling activation initiates a cascade of molecular events that modify transcription factors activity and gene expression, such as CREB, *c-fos, egr-1* (Nestler, 2001). Altogether, the present findings (reduced dopamine levels in adolescents treated with repeated ethanol) combined with previous studies (Faria et al., 2008; Soares-Simi et al., 2013) suggest a down-regulation of dopamine signaling mediated by D_1_ receptors in those mice. In other words, ethanol exposure during adolescence blunts the D1-CREB-cFos signaling stimulated by repeated ethanol. Moreover, Pascual et al. (2009) found a decreased expression of D_1_ receptors in the PFC of adolescent rats repeatedly treated with ethanol. However, we cannot discard the hypothesis that the dopamine results might be related to presynaptic effects, since dopamine D_2_ has a key role in the synthesis, release and reuptake of dopamine. Thus, both effects can co-exist to further show a decrease in dopamine signaling. In sum, different neuroplasticity pattern in the PFC could contribute to the variability in the behavioral sensitization to ethanol in adolescents, considering the role of this brain region in the phenomenon (Li et al., 1993). It is important to emphasize that the assumptions on the dopamine system signaling are limited by the fact that the analyses were performed *ex vivo*.

In addition, the low PFC dopamine content in ethanol-pretreated adult mice challenged with saline (EtOH-SAL) most likely reflects the response of a withdrawal state, reversed by an ethanol challenge injection (EtOH-EtOH).

The present results also demonstrated a main effect of age for dopamine levels in the NAc, with adolescents acutely treated with ethanol exhibiting lower levels of dopamine compared to the respective adult group. Acute exposure to ethanol (SAL-EtOH) resulted in increased NAc dopamine content in adult mice compared to their controls (SAL-SAL), which is in agreement with other studies (Di Chiara and Imperato, 1985; Peters et al., 2017). However, this effect was not evident in adolescent mice. Other studies have found decreased evoked dopamine release in the NAc of rats treated with ethanol during adolescence (Philpot et al., 2009; Zandy et al., 2015; Shnitko et al., 2016). It is important to address that these past investigations reported low ethanol-stimulated dopamine responses in distinct periods of adolescence or young adulthood. Philpot et al. (2009) detected these differences in pre and early adolescence, while Zandy et al. (2015) and Shnitko et al. (2016) treated the rats during adolescence and measured ethanol-evoked dopamine efflux during their adulthood.

Since ethanol-pretreated mice (EtOH-EtOH) showed lower Nac dopamine levels than those pretreated with saline (SAL-EtOH) in response to an ethanol challenge injection, one could suggest a dopaminergic tolerance to repeated ethanol in adults in this region. It is important to emphasize, though, that the lower dopamine responses to ethanol in the NAc may reflect a response to a withdrawal effect that was not reversed by an ethanol challenge. Indeed, reduced dopamine outflow in the NAc after withdrawal has also been previously reported (Diana et al., 1993; Schulteis et al., 1995; Karkhanis et al., 2015). Furthermore, the opposite dopaminergic responses to repeated ethanol in the PFC vs. NAc in adult mice is consistent with the evidence of the inhibitory influence of PFC on mesolimbic dopaminergic transmission (Banks and Gratton, 1995).

Compared to the PFC and NAc, striatal dopamine levels were less affected by age-dependent factors. Repeated ethanol treatment showed a trend to enhance dopamine levels in adults but not adolescents. Although behavioral sensitization is not necessarily dependent on enhanced dopamine release in the striatum (Segal and Kuczenski, 1992), the expression of sensitization reflects, at least in part, neuroadaptations in the nigrostriatal dopaminergic pathway (Kalivas and Stewart, 1991). Thus, elevated dopamine striatal levels in adults repeatedly treated with ethanol can be, at least in part, responsible for their higher sensitivity to express ethanol-induced behavioral sensitization compared to adolescents (Faria et al., 2008; Stevenson et al., 2008; Carrara-Nascimento et al., 2014; Carrara-Nascimento et al., 2017).

We chose to evaluate dopamine levels in the PFC, NAc, and striatum because of the implication of these brain regions in addictive behaviors, such as behavioral inhibitory control, motivation, drug-related hedonic effects, habit formation, and behavioral sensitization (Koob and Bloom, 1988; White, 1996; Volkow and Fowler, 2000; Berridge and Robinson, 2016). Moreover, the PFC is still under maturation during adolescence and the frontal dopaminergic system has crucial importance in motivated behaviors. Dysfunctions of PFC have been associated with impaired inhibition to self-administer a drug (Volkow et al., 2002). Our findings provide evidence that dopaminergic responses to ethanol exposure during adolescence were less intense than that induced by ethanol exposure in adults.

Drugs of abuse have the ability to disrupt the dopaminergic system and promote an unstable and dynamic state of dopamine activity, depending on the recurrent process of addiction, i.e., intoxication, withdrawal or relapse. Our data showed age-dependent differences in dopaminergic responses mainly in the PFC and NAc, with few alterations in the striatum. These regional differences may be attributed to the late ontogenic development of the PFC (for review, see Spear, 2000). NAc, in its turn, receives inputs from the PFC (Pennartz et al., 1994).

Although this study provided important age-dependent changes in the expression of behavioral sensitization and in brain regional dopamine responses to acute and repeated ethanol treatment, the neurochemical analysis was carried out 30 min after the behavioral test. This discrepancy might have implications in the direct correlations between neurochemical and behavioral effects.

A limitation of our study is that the dopamine analyses were conducted in tissue homogenates, which may not reflect a transient dopaminergic response. Although microdialysis would be more appropriate to monitor extracellular levels of dopamine over time, we aimed to investigate whether ethanol pretreatment during adolescence would change the behavioral response to ethanol and produce differences in DA levels in distinct brain regions of the reward system as a result of the lasting effects of ethanol exposure during the brain development. Despite the limitation, the age differences in dopamine and its metabolites promoted by acute or repeated ethanol in those brain regions reflect disturbances in numerous factors that can be related to synthesis, release, uptake, and metabolism of dopamine. Those results contribute to clarify differences between adolescent and adult ethanol exposure and reinforce the need for differential therapeutic approaches.

## Data Availability Statement

The datasets generated for this study are available on request to the corresponding author.

## Ethics Statement

The animal study was reviewed and approved by the Ethics Committee on Animal Use—Institute of Biomedical Sciences (#81/2013) of the Universidade de São Paulo.

## Author Contributions

PC-N and RC designed the experiments, analyzed the data and wrote the manuscript. PC-N and LH conducted the behavioral experiments. JF and CP conducted the measurement and analysis of the dopamine levels by microdialysis.

## Conflict of Interest

The authors declare that the research was conducted in the absence of any commercial or financial relationships that could be construed as a potential conflict of interest.
